# Supplementary material for: Phosphoproteomic profiling of T cell acute lymphoblastic leukemia reveals targetable kinases and combination treatment strategies
Source: Nat Commun. 2022 Feb 25;13:1048. doi: 10.1038/s41467-022-28682-1 (PMC8881579; doi:10.1038/s41467-022-28682-1)
Supplement: Supplementary file 1 — Supplementary Information [file 41467_2022_28682_MOESM1_ESM.pdf]

Supplementary Information

Phosphoproteomic Profiling of T Cell Acute Lymphoblastic Leukemia Reveals Targetable Kinases and Combination Treatment Strategies

Valentina Cordo', Mariska T. Meijer, Rico Hagelaar, Richard R. de Goeij-de Haas, Vera M. Poort, Alex A. Henneman, Sander R. Piersma, Thang V. Pham, Koichi Oshima, Adolfo A. Ferrando, Guido J.R. Zaman, Connie R. Jimenez, and Jules P.P. Meijerink

SUPPLEMENTARY TABLES

Supplementary Table 1. **Characteristics of the T-ALL cell lines used in this study.**

**Cell line**	**ATCC id**	**DSMZ id**	**ECACC id**	**Origin**	**Immuno-phenotype**	**Translocations / Fusions**	**Mutated oncogenes**	**Subgroup (rearranged oncogene (r))**
JURKAT	TIB-152	ACC-282	-	PB (Rel)	CD2+, CD3+, CD5+, CD6+, CD7+ TCRαβ+, TCRγδ-		*FBXW7, NOTCH1, TP53*	TAL/LMO
HPB-ALL	-	ACC-483	-	PB (Dx)	CD2+, CD3+, CD4+, CD5+, CD6+, CD7+, CD8+, TCRαβ+, TCRγδ-	*BCL11B-TLX3*	*FBXW7, PTEN, TP53, WT1, HRAS*	TLX (*TLX3*r)
LOUCY	CRL-2629	ACC-394	-	PB	CD2-, CD3+, CD4-, CD5+, CD +, CD7+, CD8-, TCRαβ-, TCRγδ-	*SET-NUP214*	*TP53*	ETP-ALL/immature
HSB-2	CCL-120.1	ACC-435	-	PB	CD2-, cyCD3 +, smCD3-, CD4-, CD5+, CD6+, CD7+, CD8+, TCRαβ-	*TCRB-LCK SIL-TAL1*	*NOTCH1, NRAS*	TAL/LMO (*TAL1*r)
PEER	-	ACC-6	-	PB (Rel)	CD2-, CD3+, CD4+, CD5+, CD6+, CD7+, TCRγδ+	*NUP214-ABL1 NKX2.5-BCL11B*	*NOTCH1, TP53*	ETP-ALL (*NKX2.5*r)
ALL-SIL	-	ACC-511	-	PB (Rel)	cyCD3+, CD3-, CD4+, CD5+, CD6+, CD7+, CD8+, TCRαβ-, TCRγδ-	*NUP214-ABL1*	*NOTCH1*	TLX1/NKX2.1 (*TLX1*r)
CCRF-CEM	CCL-119	ACC-240	-	PB (Rel)	CD2-, CD3+, CD4+, CD5+, CD6+, CD7 +, CD8 -		*FBXW7, NOTH1, KRAS, PIK3CA,FLT3*	TAL/LMO (*TAL1*r)
KARPAS45	-	-	6072602	BM (Dx)		*MLL-AFX*	*FBXW7, NOTCH1, TP53, WT1, JAK3, CDKN2A*	HOXA (*MLL*r)
P12-ICHIKAWA	-	ACC-34	-	PB	cyCD3+, CD4+, CD5+, CD6+, CD7+, CD8-, TCRαβ-, TCRγδ-		*FBXW7, NOTCH1, NRAS, PTEN, TP53*	TAL/LMO (*LMO2*r)
SUP-T1	CRL-1942	ACC-140	-	PE (T-LBL)	CD2+, cyCD3+, CD4+, CD5+, CD6+, CD7+,CD8+, TCRαβ-, TCRγδ-		*PHF6, PIK3CA, LCK, PTEN, RB1, TP53, JAK3*	T-LBL
MOLT16	-	ACC-29	-	PB (Rel)	CD2+, CD3+, CD4-, CD5+, CD6+, CD7+, CD8 -, CD13 -, CD19 -, CD34 -, TCRαβ+, TCRγδ-		*PIK3CA, TP53, WT1*	TAL/LMO (*LMO2*r)

Source: DSMZ, ATCC, canSAR, COSMIC, Kalender-Atak *et al.*[1], Quentmeier *et al.*[2]
Abbreviations: r, rearranged. PB, peripheral blood. BM, bone marrow. PE, pleural effusion. T-LBL, T-cell lymphoblastic lymphoma. Dx, diagnosis. Rel, relapse.

Supplementary Table 2**. Kinase inhibitors used in this study.**

Kinase inhibitor	Target	Supplier	Catalog number	CAS number
Milciclib	CDK1/2	MedChemExpress	HY-10424	802539-81-7
Dasatinib	ABL1/SFK	Sigma-Aldrich	CDS023389	302962-49-8
Imatinib mesylate	BCR-ABL1	Sigma-Aldrich	SML1027	220127-57-1
Ponatinib	ABL1/SFK	MedChemExpress	HY-12047	943319-70-8
Bosutinib	ABL1/SFK	MedChemExpress	HY-10158	380843-75-4
Nilotinib	BCR-ABL1	MedChemExpress	HY-10159	641571-10-0
A-420983	LCK	Provided by Oncolines B.V.		330789-03-2
BMS-754807	INSR/IGF-1R	MedChemExpress	HY-10200	1001350-96-4
Linsitinib (OSI-906)	INSR/IGF-1R	MedChemExpress	HY-10191	867160-71-2
GSK1904529A	INSR/IGF-1R	MedChemExpress	HY-10524	1089283-49-7
TG003	CLK1	MedChemExpress	HY-15338	719277-26-6
FRAX597	PAK	MedChemExpress	HY-15542A	1286739-19-2
Ipatasertib	AKT	MedChemExpress	HY-15186	1001264-89-6
Selumetinib	MEK1/2	Selleckchem	S1008	606143-52-6
Sirolimus	mTOR	MedChemExpress	HY-10219	53123-88-9
Ruxolitinib	JAK	Selleckchem	S1378	941678-49-5

Supplementary Fig. 1

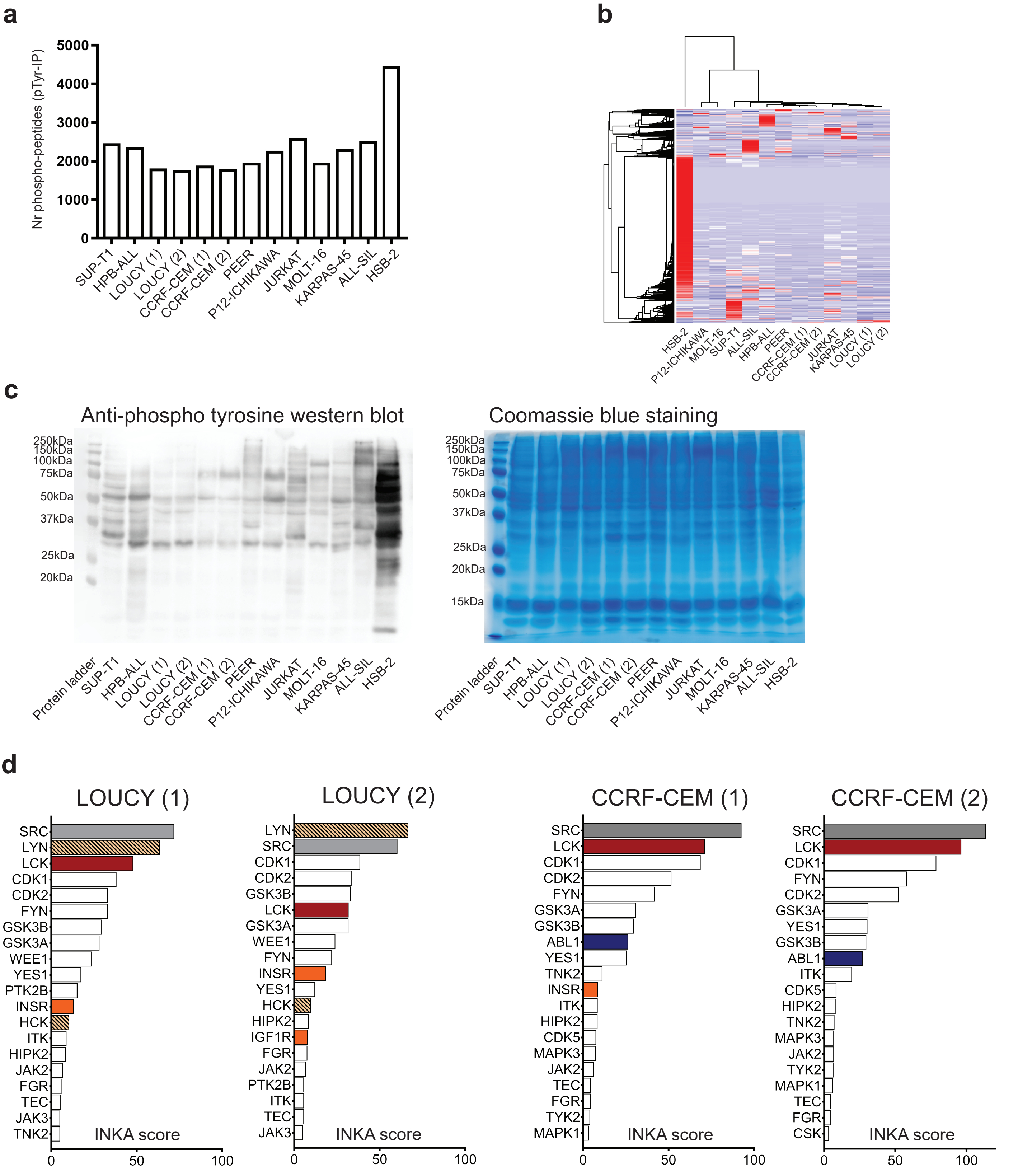

Supplementary Fig.1. **Phospho-peptides recovery and differences among T-ALL cell lines. a** Number of phospho-peptides recovered and identified in each sample following an anti-phosphorylated tyrosine immunoprecipitation. For LOUCY and CCRF-CEM, two biological replicates were used as internal reproducibility control. **b** Unsupervised clustering of phosphorylated peptides identified in each sample following an anti-phosphorylated tyrosine immunoprecipitation. **c** Anti phospho-tyrosine western blotting was performed on total lysates (30μg for each cell line) prior to phospho-peptides enrichment to evaluate the total tyrosine phosphorylation in each sample. Coomassie blue gel staining was used as a parallel loading control to assure equal protein loading. Figures are representative of two independent experiments. **d** Top20 INKA kinases inferred from the phospho-tyrosine (pY) dataset. Each bar plot illustrates the highest 20 active kinases in each cell line (biological duplicates for LOUCY and CCRF-CEM) ranked on their INKA score. Red, LCK; blue, ABL1; grey, SRC; orange, INSR/IGF-1R; striped pattern, myeloid-lineage kinases (LYN and HCK).

Supplementary Fig. 2

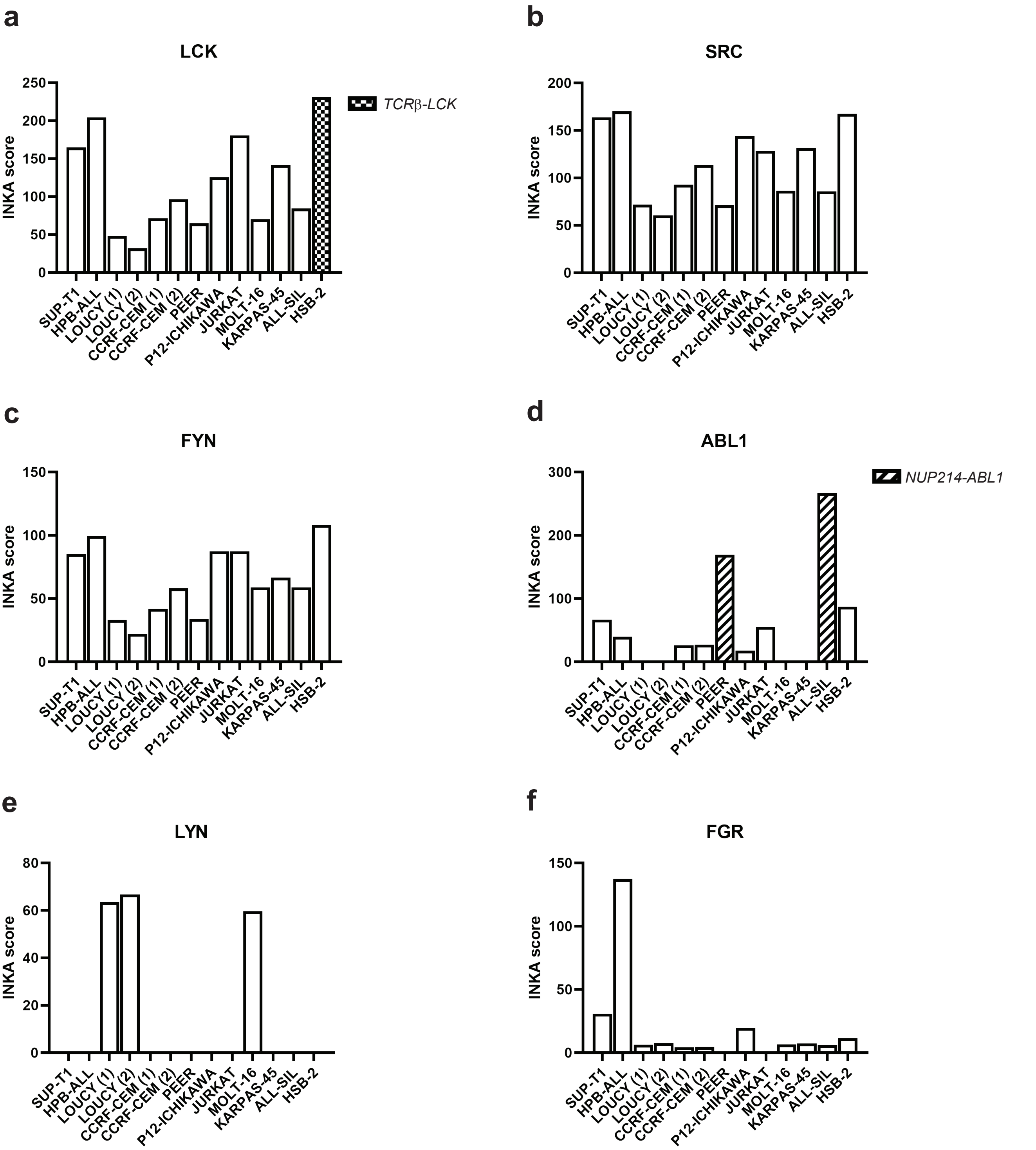

Supplementary Fig. 2. **INKA scores for several Src-family kinases (SFKs).** Each bar plot illustrates the INKA scores for different SFK members in each cell line (pY dataset). **a** LCK. The dotted pattern indicates the presence of a *TCRβ-LCK* translocation (HSB-2 cell line). **b** SRC. **c** FYN. **d** ABL1. The striped pattern indicates the presence of a *NUP214-ABL1* fusion (PEER and ALL-SIL lines). **e** LYN. **f** FGR.

Supplementary Fig. 3

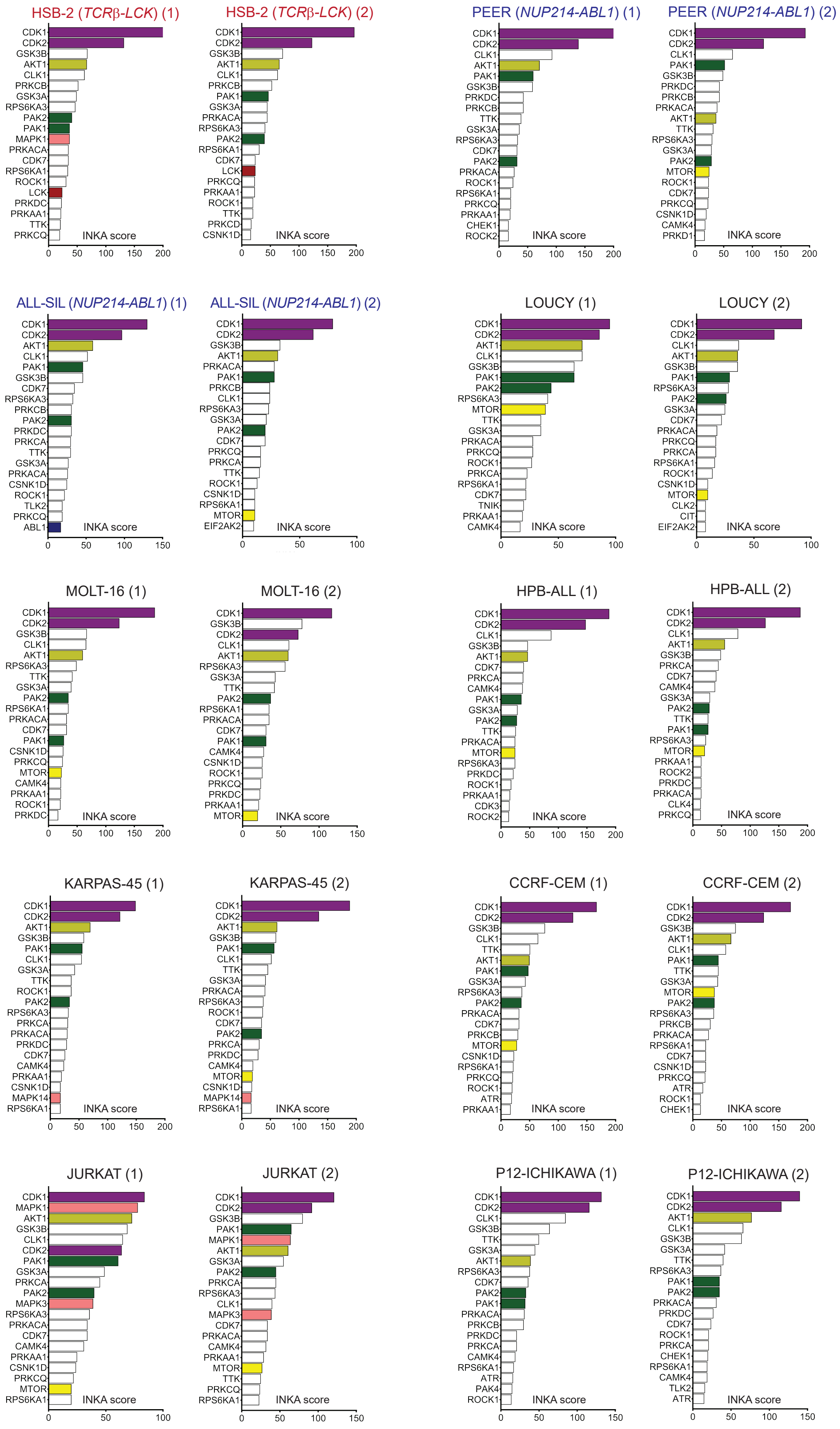

Supplementary Fig. 3. **Top20 INKA kinases inferred from the TiO_2 dataset.** Each bar plot illustrates the highest 20 active kinases in each cell line ranked on their INKA score (technical duplicates for 10 cell lines are shown). Purple, CDK1/2; dark green, PAK1/2; light green, AKT; yellow, mTOR; pink, MAPK; red, LCK, Blue: ABL1.

Supplementary Fig. 4

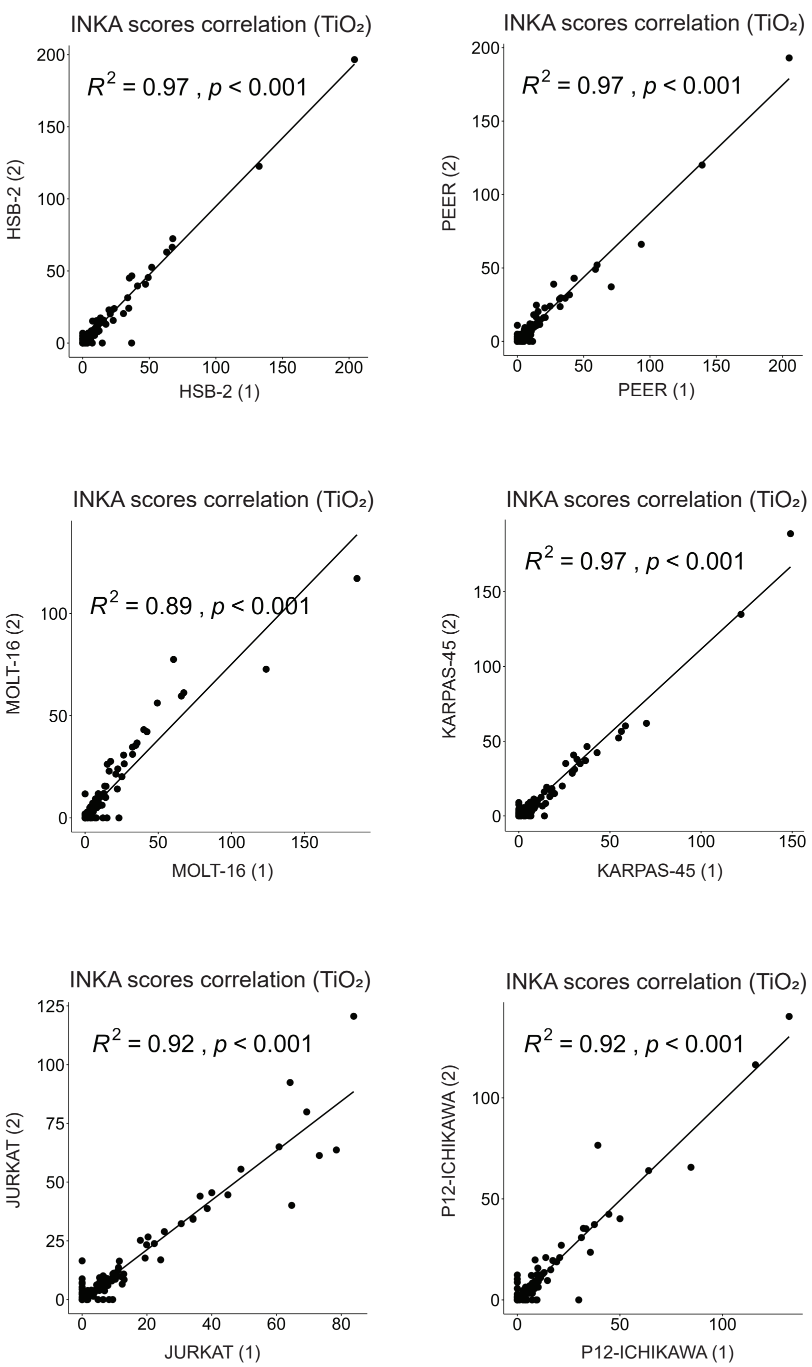

Supplementary Fig. 4. **Correlation plots TiO_2 dataset.** Each plot shows the correlation of the INKA scores between technical duplicates in the TiO_2 dataset for 8 cell lines (Pearson's correlation, two-sided t-test, $p < 0.001$).

Supplementary Fig. 5

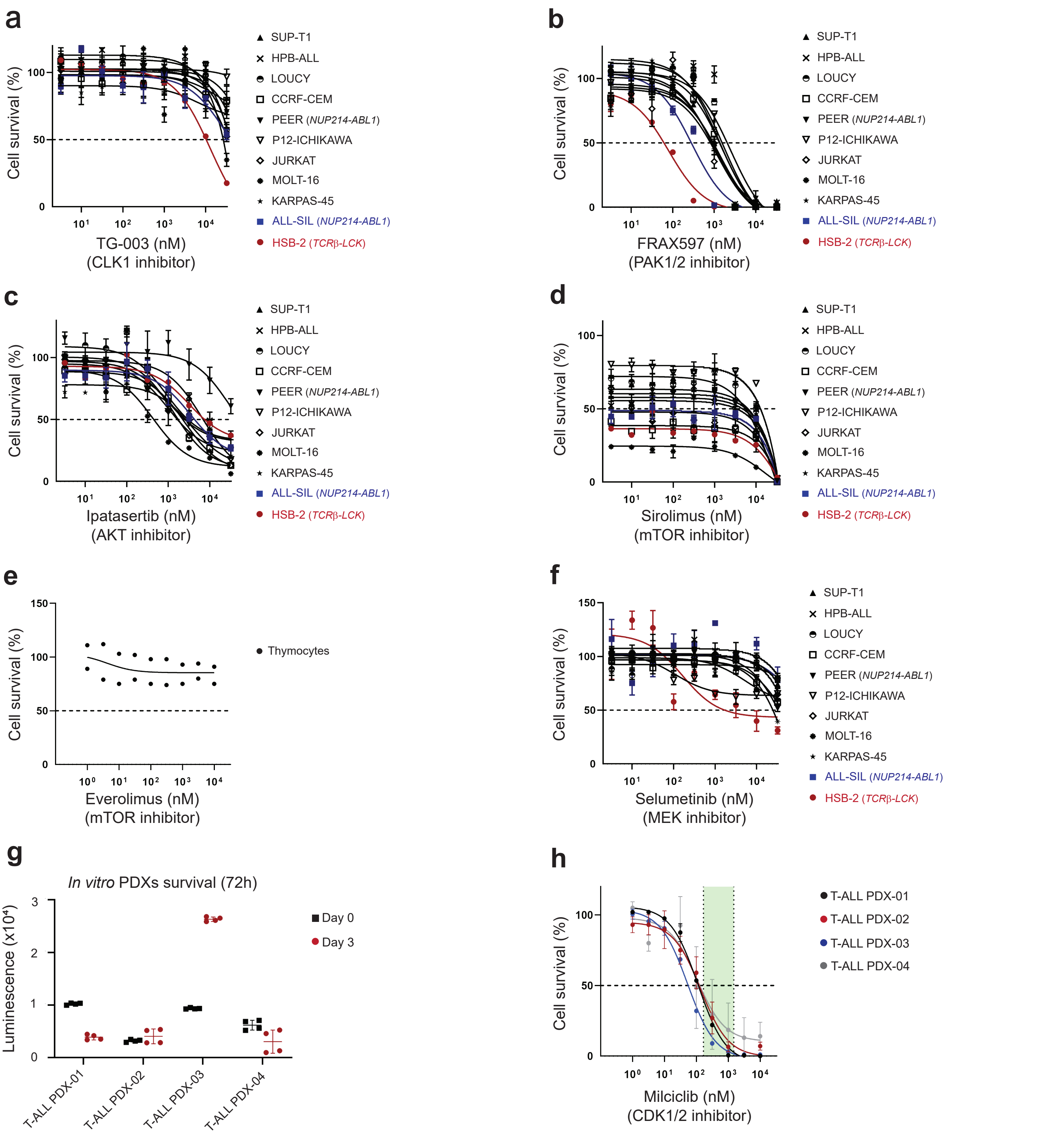

Supplementary Fig. 5. **Dose-response curves of kinase inhibitors (CLK1i, PAKi, AKTi, mTORi, MEKi, CDKi).** Dose-response curves of kinase inhibitors treatment. Cell lines were treated with increasing concentrations of the CLK1 inhibitor TG-003 (**a**), PAK inhibitor FRAX597 (**b**), AKT inhibitor ipatasertib (**c**), mTOR inhibitor sirolimus (**d**) in a 3.2nM–32µM range in triplicate and cell viability was assessed after 72 hours using the colorimetric MTT assay. Cell survival was calculated in comparison to the untreated control. Each point represents the mean and standard deviation of the triplicate. **e** Dose-response curves of everolimus treatment *ex vivo* in healthy thymocytes isolated from a pediatric thymic biopsy. Cells were treated with increasing concentrations (1nM–10µM) of everolimus in duplicates. Cell survival was calculated in comparison to the untreated control (DMSO only). **f** Dose-response curves of selumetinib treatment in T-ALL cell lines. Each point represents the mean and standard deviation of the triplicate. **g** *In vitro* survival of blasts obtained from four T-ALL patient-derived murine xenografts (PDXs). Luminescence signal recorded at day 0 (time of seeding) and day 3 (72h culture). Symbols indicate replicates per conditions (N = 4), the horizontal line the mean, and the error bars indicate the average of the mean. **h** Dose-response curves of milciclib treatment *ex vivo* in 4 PDXs. Cells were treated with increasing concentrations of milciclib (1nM-10µM) for 72 hours and the survival was calculated in comparison to the untreated control (DMSO only). The green box indicates the clinical concentration range of milciclib. Each point represents the mean and standard deviation of the duplicate.

Supplementary Fig. 6

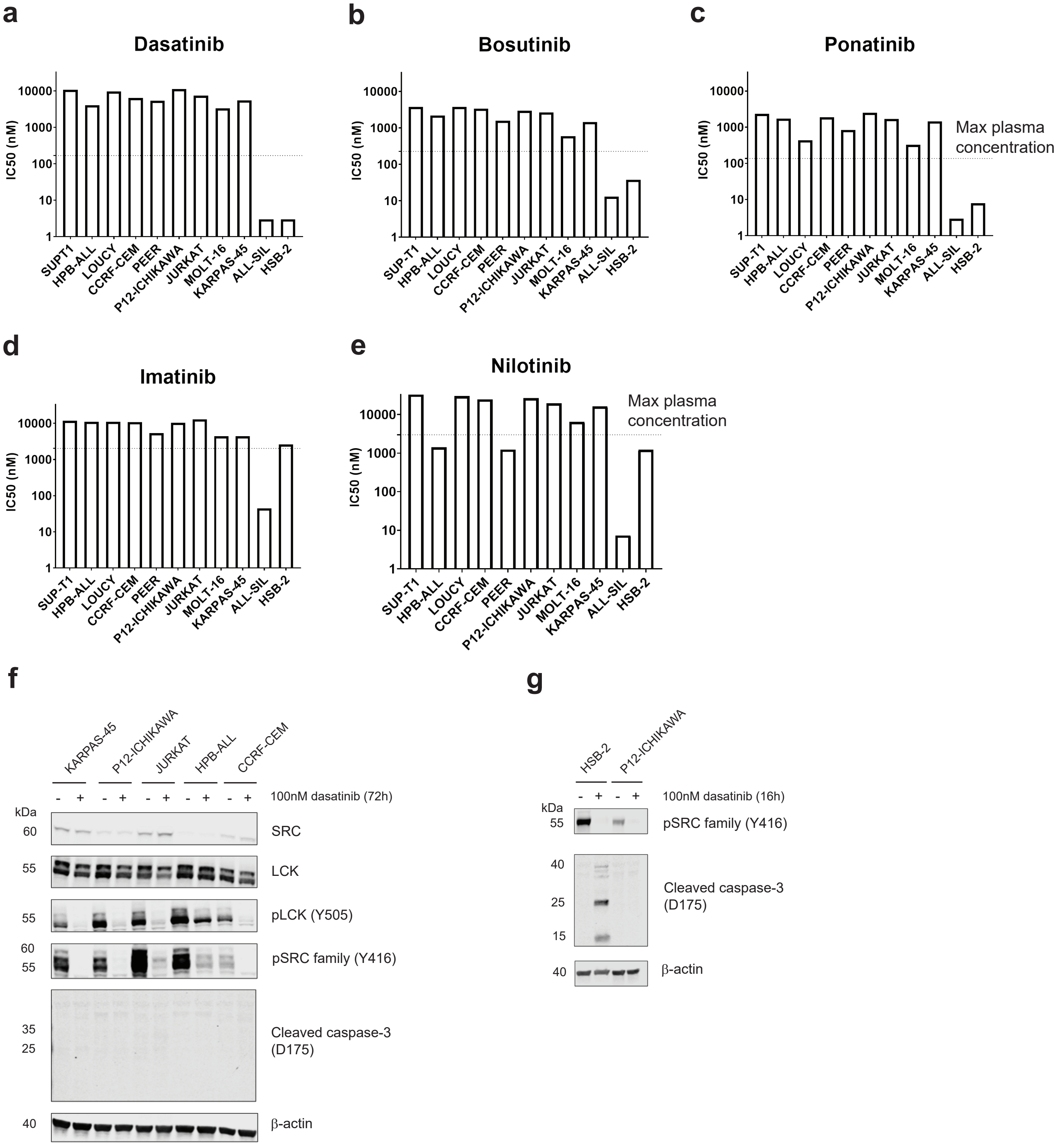

Supplementary Fig. 6. **IC₅₀ values for different kinase inhibitors tested in T-ALL cell lines and *in vitro* SFKs inhibition.** Each bar plot shows the IC_{50} values for each cell line for the SFKs/ABL inhibitors tested. The dotted line indicates the highest plasma concentration achieved in patients. **a** dasatinib, **b** bosutinib, **c** ponatinib, **d** imatinib, **e** nilotinib. **f** Western blot analysis upon dasatinib treatment *in vitro*. Cell lines expressing high levels of LCK and/or SRC were treated with 100nM dasatinib for 72 hours. The image is representative of two independent experiments. **g** Western blot analysis upon dasatinib treatment *in vitro*. A dasatinib-sensitive cell line (HSB-2) and a dasatinib-resistant cell line (P12-ICHIKAWA) were treated with 100nM dasatinib for 16 hours. The image is representative of two independent experiments.

Supplementary Fig. 7

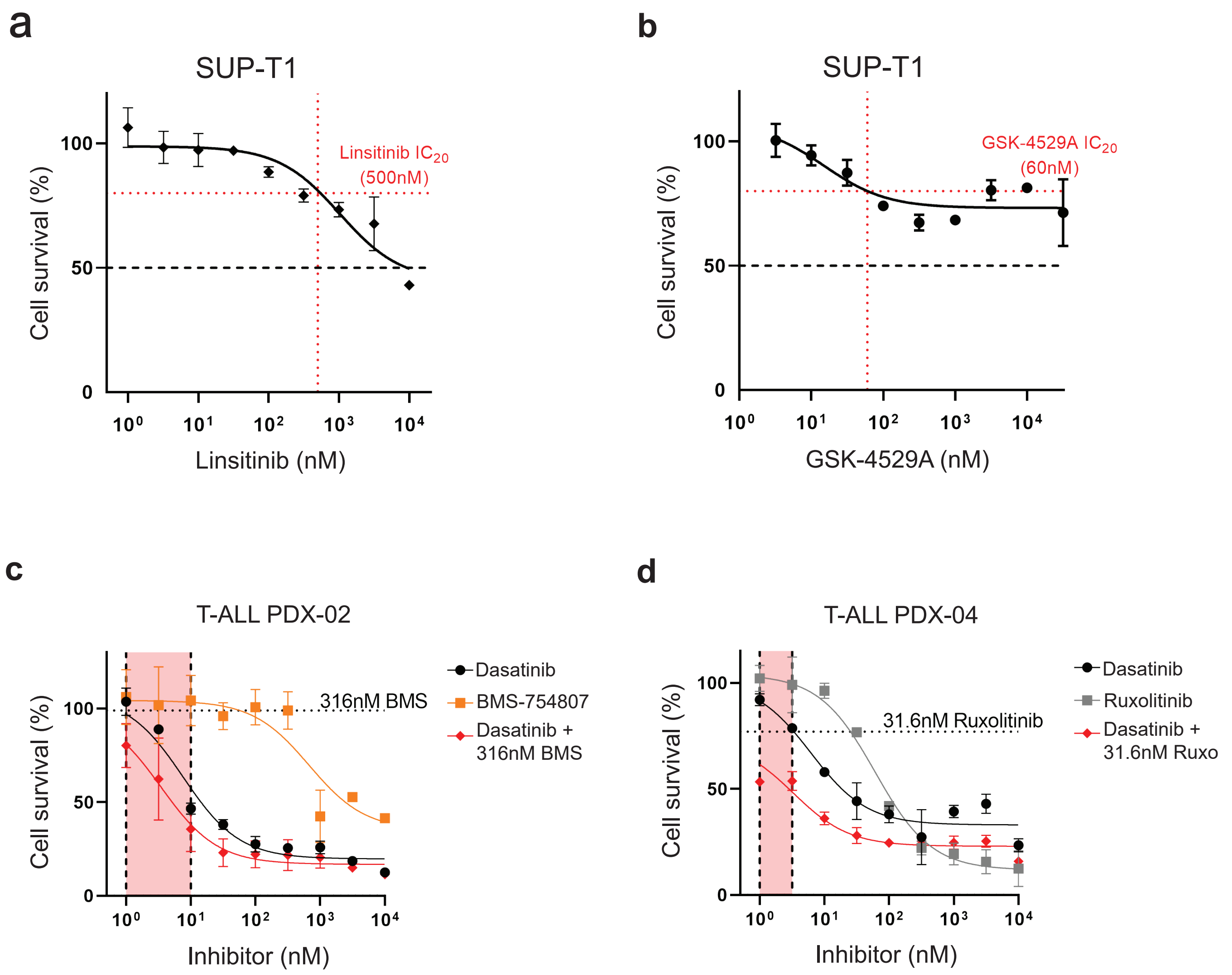

Supplementary Fig. 7. **Dose-response curves of the INSR/IGF-1R inhibitors linsitinib and GSK-4529A in SUP-T1 cells and combination treatment *ex vivo* in PDXs.** Dose-response curves of linsitinib (**a**) and GSK-4529A (**b**) in SUP-T1 cells. Cells were treated for 72 hours with increasing concentrations of linsitinib (1nM–10µM range) or GSK-4529A (3.2nM–32µM range) as single treatment. Cell survival was calculated in comparison to the untreated control. Each point represents the mean and standard deviation of the triplicate. **c** Dose-response curves of dasatinib, BMS-754807 and combination treatment *ex vivo* in PDX-02. Cells were treated for 72 hours and viability was calculated in relation to untreated control cells (DMSO only). Each point represents the mean and standard deviation of the duplicate. The red box indicates the area of synergy of the drug matrix. **d** Dose-response curves of dasatinib, ruxolitinib and combination treatment *ex vivo* in PDX-04. Cells were treated for 72 hours and viability was calculated in relation to untreated control cells (DMSO only). Each point represents the mean and standard deviation of the duplicate. The red box indicates the area of synergy of the drug matrix.

Supplementary Fig. 8

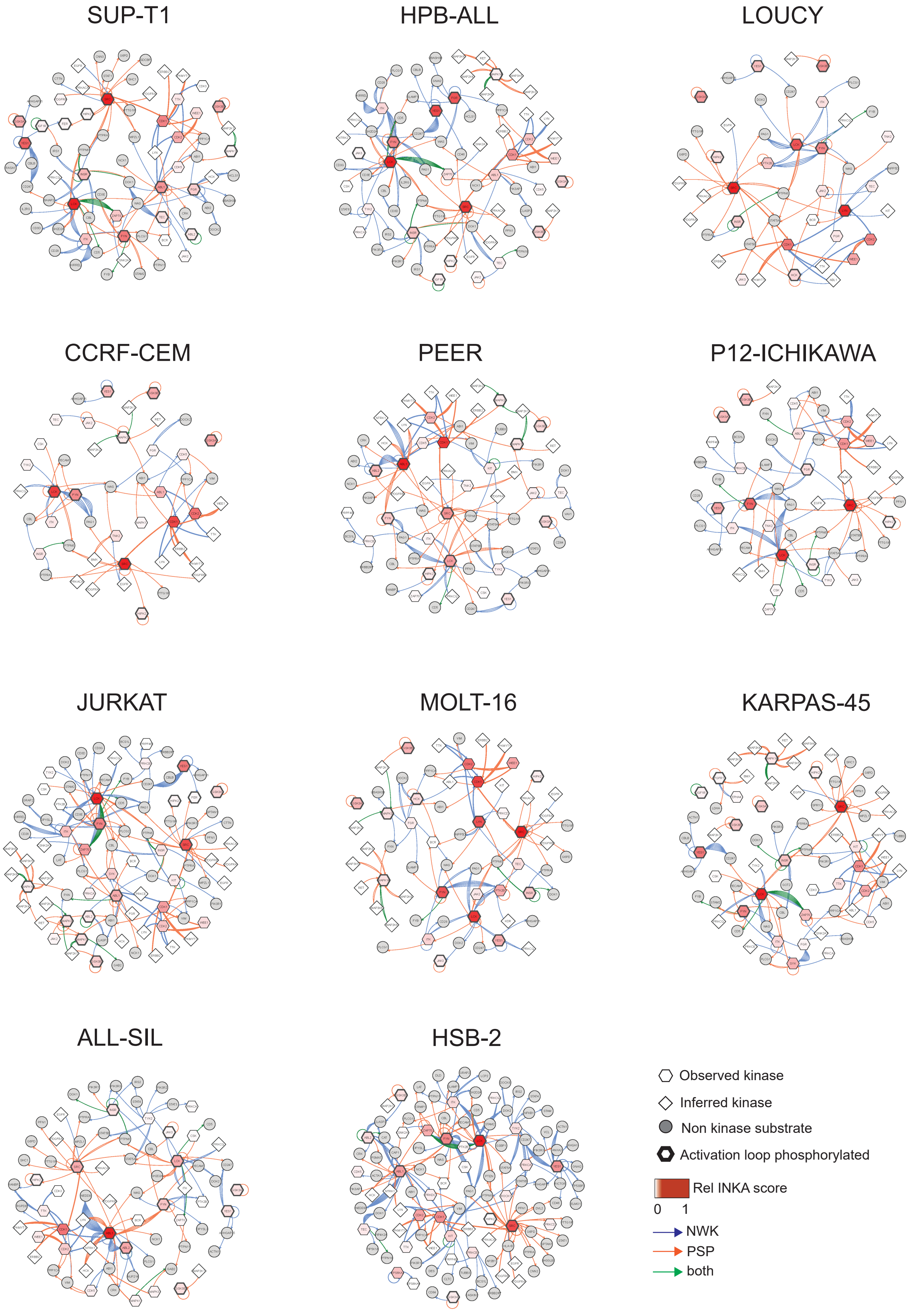

Supplementary Fig. 8. **Kinase substrate-networks.** Each graph represents the main kinase-substrate relation networks inferred from the phosphoproteomic data (pY dataset) by INKA analysis for each T-ALL cell line.

Supplementary Fig. 9

a

Gating used for Figure 2b
Cell cycle analysis

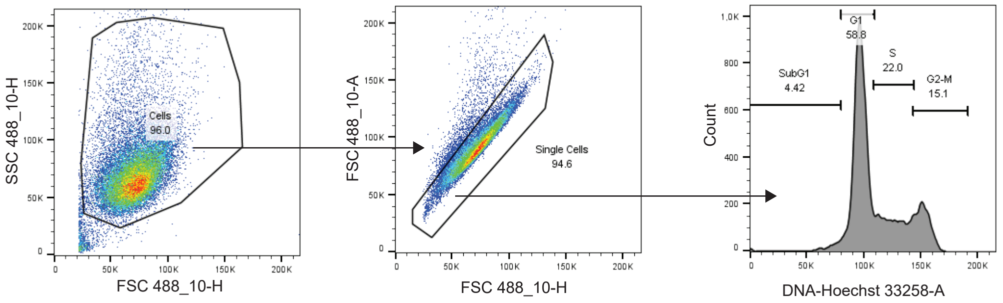

b

Gating used for Figure 2c
Annexin V / PI staining of apoptotic cells

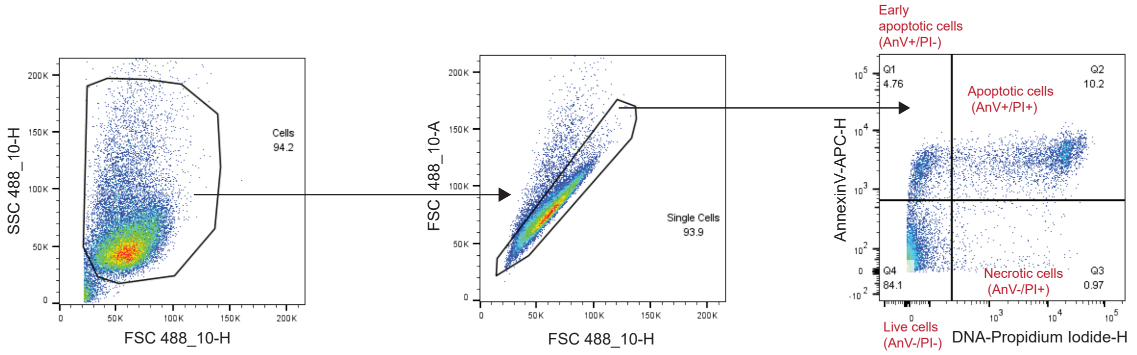

Supplementary Fig. 9. **Gating strategy for FACS analysis of cell cycle and apoptosis (Figures 2b and 2c). a** The first gating is made to remove debris based on size, then the selected cellular population is gated again to remove duplets, and eventually the phases of the cell cycle are defined based on the peaks in the distribution of the Hoechst (UV) signal intesity (histogram). Processed data are shown in Figure 2b. **b** The first gating is made to remove debris based on size, then the selected cellular population is gated to remove duplets, and eventually gated cells are defined as live (Annexin V-/PI-), necrotic (Annexin V-/PI+), early apoptotic (Annexin V+/PI-), and apoptotic (Annexin V+/PI+). Processed data are shown in Figure 2c where apoptotic cells are defined as the sum of early apoptotic (Annexin V+/PI-) and apoptotic (Annexin V+/PI+) cells.

SUPPLEMENTARY REFERENCES

1 Kalender Atak, Z. *et al.* High accuracy mutation detection in leukemia on a selected panel of cancer genes. *PLoS One* **7**, e38463, doi:10.1371/journal.pone.0038463 (2012).

2 Quentmeier, H. *et al.* The LL-100 panel: 100 cell lines for blood cancer studies. *Sci Rep* **9**, 8218, doi:10.1038/s41598-019-44491-x (2019).
