# Supplementary material for: Phosphoproteomic profiling of T cell acute lymphoblastic leukemia reveals targetable kinases and combination treatment strategies
Source: Nat Commun. 2022 Feb 25;13:1048. doi: 10.1038/s41467-022-28682-1 (PMC8881579; doi:10.1038/s41467-022-28682-1)
Supplement: Supplementary file 3 — Description of Additional Supplementary Files [file 41467_2022_28682_MOESM3_ESM.pdf]

Description of Additional Supplementary Files

File name: Supplementary Data 1

Description: List of somatic mutations identified by whole-exome sequencing in the 4 patient-derived xenografts used in this study (Figure 6).
