# Supplementary material for: Phosphoproteomic profiling of T cell acute lymphoblastic leukemia reveals targetable kinases and combination treatment strategies
Source: Nat Commun. 2022 Feb 25;13:1048. doi: 10.1038/s41467-022-28682-1 (PMC8881579; doi:10.1038/s41467-022-28682-1)
Supplement: Supplementary file 5 — Reporting summary [file 41467_2022_28682_MOESM5_ESM.pdf]

nature portfolio

Corresponding author(s): Jules P.P. Meijerink
Last updated by author(s): Jan 4, 2022

Reporting Summary

Nature Portfolio wishes to improve the reproducibility of the work that we publish. This form provides structure for consistency and transparency in reporting. For further information on Nature Portfolio policies, see our Editorial Policies and the Editorial Policy Checklist.

Statistics

For all statistical analyses, confirm that the following items are present in the figure legend, table legend, main text, or Methods section.

n/a	Confirmed	
☐	☒	The exact sample size (*n*) for each experimental group/condition, given as a discrete number and unit of measurement
☒	☐	A statement on whether measurements were taken from distinct samples or whether the same sample was measured repeatedly
☐	☒	The statistical test(s) used AND whether they are one- or two-sided<br>*Only common tests should be described solely by name; describe more complex techniques in the Methods section.*
☒	☐	A description of all covariates tested
☐	☒	A description of any assumptions or corrections, such as tests of normality and adjustment for multiple comparisons
☐	☒	A full description of the statistical parameters including central tendency (e.g. means) or other basic estimates (e.g. regression coefficient) AND variation (e.g. standard deviation) or associated estimates of uncertainty (e.g. confidence intervals)
☐	☒	For null hypothesis testing, the test statistic (e.g. *F*, *t*, *r*) with confidence intervals, effect sizes, degrees of freedom and *P* value noted<br>*Give P values as exact values whenever suitable.*
☒	☐	For Bayesian analysis, information on the choice of priors and Markov chain Monte Carlo settings
☒	☐	For hierarchical and complex designs, identification of the appropriate level for tests and full reporting of outcomes
☐	☒	Estimates of effect sizes (e.g. Cohen's *d*, Pearson's *r*), indicating how they were calculated

Our web collection on statistics for biologists contains articles on many of the points above.

Software and code

Policy information about availability of computer code

Data collection	Mass spectrometry data were collected using an Ultimate 3000 nanoLC-MS/MS system (Thermo Fisher) coupled to a Q Exactive HF mass spectrometer (Thermo Fisher).<br><br>Cell cycle profiles and annexin V/propidium iodide stainings were acquired using a ZE5 flow cytometer (BioRAD).
Data analysis	For protein identification, MS/MS spectra were searched against theoretical spectra from the UniProt complete human proteome FASTA file (release January 2018, 42,258 entries) using the MaxQuant 1.6.0.16 software.<br>Inferred kinase activity was calculated using the INKA pipeline. The latest version of the INKA code is avalilable online at https://inkascore.org/.<br><br>Dose-response curves and statistical analyses were generated using the GraphPad Prism 9.0.1 software (Prism). FACS data were analyzed using the FlowJo v10.7.1 software (FlowJo). Drug synergy was calculated using RStudio (version 1.3) with the SynergyFinder R package (version 2.4.16). The figures have been prepared using Adobe Illustrator 2021 (version 25.0.0).

For manuscripts utilizing custom algorithms or software that are central to the research but not yet described in published literature, software must be made available to editors and reviewers. We strongly encourage code deposition in a community repository (e.g. GitHub). See the Nature Portfolio guidelines for submitting code & software for further information.

Data

Policy information about availability of data

All manuscripts must include a data availability statement. This statement should provide the following information, where applicable:

- Accession codes, unique identifiers, or web links for publicly available datasets
- A description of any restrictions on data availability
- For clinical datasets or third party data, please ensure that the statement adheres to our policy

The mass spectrometry proteomics data have been deposited to the ProteomeXchange Consortium via the PRIDE partner repository with the dataset identifier PXD024807. The human Swiss-Prot database used for raw data search was downloaded from the UniProt database [https://www.uniprot.org/]. The AML phosphoproteomic data used in Fig. 1e was downloaded from van Alphen et al. (ref #22). The targets of milciclib were identified browsing the ProteomicsDB database (refs #28,29) [https://www.proteomicsdb.org/]. Source data are provided with this paper.

Field-specific reporting

Please select the one below that is the best fit for your research. If you are not sure, read the appropriate sections before making your selection.

☒ Life sciences ☐ Behavioural & social sciences ☐ Ecological, evolutionary & environmental sciences

For a reference copy of the document with all sections, see nature.com/documents/nr-reporting-summary-flat.pdf

Life sciences study design

All studies must disclose on these points even when the disclosure is negative.

Sample size	No statistical method was used to determine the sample size. For the mass spectrometry-based phosphoproteomic profiling, 11 cell lines were chosen to include different disease subtypes and known genomic abnormalities representative of T-ALL, based on the authors experience. For validation experiments, 3 biological independent samples were used to achieve sufficient power for statistical analyses.
Data exclusions	No data were excluded.
Replication	The experiments were performed in duplicate/triplicate for each condition. Results were confirmed in at least two independent experiments, unless specified otherwise in the figure legends.
Randomization	Allocation of cell culture flasks/plate wells to treatment or vehicle was random. However, no randomization was performed for the data acquisition to limit the chance of human errors during the process.
Blinding	Blinding was not possible (experimental set-up and data acquisition was performed by the same person).

Reporting for specific materials, systems and methods

We require information from authors about some types of materials, experimental systems and methods used in many studies. Here, indicate whether each material, system or method listed is relevant to your study. If you are not sure if a list item applies to your research, read the appropriate section before selecting a response.

Materials & experimental systems

n/a	Involved in the study	
☐	☒	Antibodies
☐	☒	Eukaryotic cell lines
☒	☐	Palaeontology and archaeology
☐	☒	Animals and other organisms
☐	☒	Human research participants
☒	☐	Clinical data
☒	☐	Dual use research of concern

Methods

n/a	Involved in the study	
☒	☐	ChIP-seq
☐	☒	Flow cytometry
☒	☐	MRI-based neuroimaging

Antibodies

Antibodies used	Western Blotting:<br>anti-P-Tyr-1000 (Cell Signaling Technology cat# 8954)<br>anti-Lck (Cell Signaling Technology cat# 2752)<br>anti-Src L4A1 (Cell Signaling Technology cat# 2110)<br>anti-phospho Lck (Tyr505) (Cell Signaling Technology cat# 2751)<br>anti-phospho Src Family (Tyr416) (Cell Signaling Technology cat# 2101)

anti-IGF1Rβ (Cell Signaling Technology cat# 3027)
anti-phospho IGF1Rβ (Y1135) (Cell Signaling Technology cat# 3918)
anti-phospho mTOR (S2448) (Cell Signaling Technology cat# 2971)
anti-phospho p70 S6K (T421/S424) (Cell Signaling Technology cat# 9204)
anti-AKT (Cell Signaling Technology cat# 9272)
anti-phospho AKT (S473) (Cell Signaling Technology cat# 9271)
anti-p44-42 MAPK (ERK1/2) (137F5) (Cell Signaling Technology cat# 4695)
anti-phospho p44-42 MAPK (T202/Y204) (D13.14.4E) (Cell Signaling Technology cat# 4370)
anti-cleaved caspase-3 (Asp175) (Cell Signaling Technology cat# 9661)
anti-β actin (Abcam, cat# ab6276)

Phospho-peptides enrichment:
PTMScan® Phospho-Tyrosine Rabbit mAb (P-Tyr-1000) Kit (Cell Signaling Technology cat# 8803)

Flow cytometry:
Annexin V-APC antibody (Biolegend cat# 640920)

Validation

No validation was performed since all the antibodies used in this study were validated by the vendor and reported in the antibody datasheet, as indicated below:

Western Blotting:
anti-P-Tyr-1000 (Cell Signaling Technology cat# 8954): https://www.cellsignal.com/products/primary-antibodies/phospho-tyrosine-p-tyr-1000-multimab-rabbit-mab-mix/8954
anti-Lck (Cell Signaling Technology cat# 2752): https://www.cellsignal.com/products/primary-antibodies/lck-antibody/2752
anti-Src L4A1 (Cell Signaling Technology cat# 2110): https://www.cellsignal.com/products/primary-antibodies/src-l4a1-mouse-mab/2110
anti-phospho Lck (Tyr505) (Cell Signaling Technology cat# 2751): https://www.cellsignal.com/products/primary-antibodies/phospho-lck-tyr505-antibody/2751
anti-phospho Src Family (Tyr416) (Cell Signaling Technology cat# 2101): https://www.cellsignal.com/products/primary-antibodies/phospho-src-family-tyr416-antibody/2101
anti-IGF1Rβ (Cell Signaling Technology cat# 3027): https://www.cellsignal.com/products/primary-antibodies/igf-i-receptor-b-antibody/3027
anti-phospho IGF1Rβ (Y1135) (Cell Signaling Technology cat# 3918): https://www.cellsignal.com/products/primary-antibodies/phospho-igf-i-receptor-b-tyr1135-da7a8-rabbit-mab/3918
anti-phospho mTOR (S2448) (Cell Signaling Technology cat# 2971): https://www.cellsignal.com/products/primary-antibodies/phospho-mtor-ser2448-antibody/2971
anti-phospho p70 S6K (T421/S424) (Cell Signaling Technology cat# 9204): https://www.cellsignal.com/products/primary-antibodies/phospho-p70-s6-kinase-thr421-ser424-antibody/9204
anti-AKT (Cell Signaling Technology cat# 9272): https://www.cellsignal.com/products/primary-antibodies/akt-antibody/9272
anti-phospho AKT (S473) (Cell Signaling Technology cat# 9271): https://www.cellsignal.com/products/primary-antibodies/phospho-akt-ser473-antibody/9271
anti-p44-42 MAPK (ERK1/2) (137F5) (Cell Signaling Technology cat# 4695): https://www.cellsignal.com/products/primary-antibodies/p44-42-mapk-erk1-2-137f5-rabbit-mab/4695
anti-phospho p44-42 MAPK (T202/Y204) (D13.14.4E) (Cell Signaling Technology cat# 4370): https://www.cellsignal.com/products/primary-antibodies/phospho-p44-42-mapk-erk1-2-thr202-tyr204-d13-14-4e-xp-rabbit-mab/4370
anti-cleaved caspase-3 (Asp175) (Cell Signaling Technology cat# 9661) https://www.cellsignal.com/products/primary-antibodies/cleaved-caspase-3-asp175-antibody/9661
anti-β actin (Abcam, cat# ab6276): https://www.abcam.com/beta-actin-antibody-ac-15-ab6276.html

Phospho-peptides enrichment:
PTMScan® Phospho-Tyrosine Rabbit mAb (P-Tyr-1000) Kit (Cell Signaling Technology cat# 8803):https://www.cellsignal.com/products/proteomic-analysis-products/phospho-tyrosine-rabbit-mab-p-tyr-1000-kit/8803

Flow cytometry:
Annexin V-APC antibody (Biolegend cat# 640920): https://www.biolegend.com/en-us/products/apc-annexin-v-8144

Eukaryotic cell lines

Policy information about cell lines

Cell line source(s)

The following cell lines were purchased from DSMZ (Germany) or ATCC (USA):
JURKAT, HPB-ALL, LOUCY, HSB-2, PEER, ALL-SIL, CCRF-CEM, KARPAS45, P12- ICHIKAWA, SUP-T1, MOLT16.

Authentication

Cell lines authentication was performed via short tandem repeat (STR) profiling.

Mycoplasma contamination	Cells were periodically tested for the absence of mycoplasma contamination using the MycoAlert Mycoplasma Detection Kit (Lonza cat# LT07-118). All the cell lines used in this study tested negative for mycoplasma contamination.
Commonly misidentified lines (See ICLAC register)	No commonly misidentified line was used in this study.

Animals and other organisms

Policy information about studies involving animals; ARRIVE guidelines recommended for reporting animal research

Laboratory animals	NOD/scid/Gamma (NOD.Cg-Prkdcscid Il2rgtm1Wjl/SzJ) female mice of 8-10 weeks of age (Charles River Laboratories, France) were used to generate patient-derived xenografts.
Wild animals	The study did not involve wild animals.
Field-collected samples	The study did not involve samples collected from the field.
Ethics oversight	Animal experiments were approved by the Animal Welfare Committee of the Princess Máxima Center for pediatric oncology (Utrecht, the Netherlands) and were carried out at the animal facility of the Hubrecht Institute (Utrecht, the Netherlands) under specific pathogen-free conditions and in accordance with animal welfare, FELASA (Federation of European Laboratory Animal Science Associations), ethical and institutional guidelines.

Note that full information on the approval of the study protocol must also be provided in the manuscript.

Human research participants

Policy information about studies involving human research participants

Population characteristics	Pediatric subjects (both male and female) of 0-12 years of age (children older than 12 years were excluded since they present a thymus with reduced cellularity due to the organ regression during puberty and adulthood) who need a surgical cardiac operation to correct a congenital heart defect. In order to access the cardiac area, the thymus (or part of the organ) is removed and used for research purposes. Only leftover material (not used for any diagnostic purpose) from the surgical operation was used for research purposes. No other clinical data (treatment, outcome...) was acquired since it was not relevant for the purpose of our study.
Recruitment	All patients younger than 12 years of age whose legal guardians provided signed informed consent were included if leftover material was collected during the surgical cardiac operation and not used for any diagnostic purpose. The nature of the thymic organ and the related age-associated thymic involution requires the selection of young subjects to obtain sufficient and funtional thymocytes/ t-cell precursors.
Ethics oversight	The study protocol was approved by the ethical committee and the biobank of the Utrecht university medical center (TCbio-18-181).

Note that full information on the approval of the study protocol must also be provided in the manuscript.

Flow Cytometry

Plots

Confirm that:

- [x] The axis labels state the marker and fluorochrome used (e.g. CD4-FITC).
- [x] The axis scales are clearly visible. Include numbers along axes only for bottom left plot of group (a 'group' is an analysis of identical markers).
- [x] All plots are contour plots with outliers or pseudocolor plots.
- [x] A numerical value for number of cells or percentage (with statistics) is provided.

Methodology

Sample preparation	Human T cell acute lymphoblastic leukemia cells from established cell lines were used. For cell cycle analysis, 200,000 live cells per condition were stained with Hoechst (7.5µg/ml) for 1 hour at 37ºC and then incubated for 15 minutes on ice before FACS analysis. For Annexin V/propidium iodide (PI) staining of apoptotic cells, 200,000 live cells were stained with Annexin V-APC antibody (Biolegend cat# 640920) diluted 1:20 in Annexin V-binding buffer (Invitrogen cat# V13246) for 15 minutes at room temperature (RT) in the dark. PI (Miltenyi) was added at a final concentration of 0.5µg/ml just before FACS measurement.
Instrument	ZE5 flow cytometer (BioRAD).
Software	FlowJo v10.7.1.
Cell population abundance	For cell cycle analysis, the percentage of cells in subG1, G1, S, and G2-M is indicated (30,000 events recorded). Apoptotic cells are defined as the sum of annexin V-APC positive cells and propidium iodide positive cells.

Gating strategy	Cells were gated based on size to exclude debris (FSC/SSC). Subsequently, from the selected population, single cells were gated (FSC-H/FSC-A). For cell cycle analysis, the Hoechst histogram was used to determine the % of cells in subG1, G1, S, and G2-M, respectively.

☒ Tick this box to confirm that a figure exemplifying the gating strategy is provided in the Supplementary Information.
